# Beyond the variant: hereditary cancer awareness in the multi-omics era

**DOI:** 10.1186/s41065-026-00738-y

**Published:** 2026-09-28

**Authors:** Julhash U Kazi, Ramin Massoumi

**Affiliations:** Division of Translational Cancer Research, Department of Laboratory Medicine, Lund University, Lund, 22363 Sweden

## Abstract

Hereditary cancer awareness has entered a new phase. For decades, awareness has focused on recognizing familial risk, identifying pathogenic germline variants, and helping families access counselling, surveillance and prevention. Yet in 2026, the main challenge is no longer only whether a variant can be detected, but how inherited risk can be interpreted, communicated and translated into action in the biological context of each tissue and tumor. The recent publication landscape of *Hereditas* illustrates this shift: cancer genetics is now inseparable from RNA regulation, epigenetics, metabolism, immune context, cellular plasticity, therapy resistance, computational modelling and precision intervention. This Editorial argues that hereditary cancer awareness must move beyond the variant without moving away from the familial risk. One step in this direction could be to use multi-omics as a bridge between genetic risk and precision prevention.

## Main text

Last year, we argued that *Hereditas* has a responsibility to lead hereditary cancer awareness because genetics is never only about molecules; it is about people, families and futures [1]. That responsibility remains. However, the scientific language of inherited cancer risk is changing. A pathogenic germline variant in *BRCA1*, *MLH1*, *APC*, *TP53*, *PTEN* or another cancer-predisposition gene may begin a clinical conversation, but it rarely ends it [2, 3]. Families ask what the result means for the individual, for relatives, for children, for screening, and for treatment, if cancer develops. Clinicians ask about penetrance, tumor spectrum, modifier effects, variant interpretation and therapeutic vulnerability. Researchers ask why the same inherited defect can produce different cancers, different ages of onset, and different responses to therapy. These questions cannot be answered by DNA sequence alone. Nor can they be answered by awareness campaigns that present hereditary cancer as a simple binary of carrier or non-carrier. The lived reality is more complex: one result may matter to many relatives, but each relative will experience that information at different ages, with different exposures, reproductive choices, comorbidities, and health-system barriers.

The phrase “beyond the variant” is therefore not a retreat from germline genetics. It is an expansion of hereditary cancer awareness into functional interpretation. A variant becomes meaningful when we understand the regulatory circuits, cellular states and tissue environments through which inherited susceptibility becomes disease. This is where multi-omics now matters, not as a fashionable label but as a practical framework for connecting inherited predisposition to measurable biology. Genomics identifies risk; transcriptomics reveals whether pathways are activated; epigenomics explains chromatin context and dosage; proteomics and post-translational biology show functional consequences; metabolomics captures altered cellular needs; and single-cell and spatial approaches reveal which cells carry, express, suppress or exploit these changes [4, 5]. Awareness in the multi-omics era should help patients and families understand not only “what variant do I carry?” but also “what biological opportunities exist for prevention, surveillance and treatment?”

Recent studies published in *Hereditas* offer an overview of this rapidly developing area. Several studies highlight the regulatory genome as a major interface between cancer risk and cancer behavior. Work on miR-557 in cervical cancer showed how a microRNA can suppress malignant phenotypes by targeting TAOK1 and modulating ferroptosis-associated features [6]. Complementary studies reported oncogenic roles for miR-1229-3p in cervical cancer through FBXL5 and EMT, miR-4513 in non-small cell lung cancer through VIPR1, and miR-3913-3p in lung adenocarcinoma through STX3 [7–9]. These are not hereditary cancer studies in the narrow sense, but they are highly relevant to hereditary cancer awareness because they remind us that cancer predisposition is interpreted through regulatory networks. The inherited genome sets risk; the expressed and regulated genome shapes behavior.

Long non-coding RNAs, snoRNAs and miRNA-mRNA axes further extend this message. In hepatocellular carcinoma, MIR9-3HG was reported to promote progression through a has-let-7c-5p/THBS1 ceRNA axis, while an integrated analysis identified a miR-125b-2/UCK2 axis linked to extracellular matrix and growth-factor signaling [10, 11]. Another HCC study connected folate-driven changes to snoRNA function, ribosomal biology and epigenetic regulation [12]. A prognostic signature based on myeloid-derived suppressor cell-related lncRNAs linked non-coding RNA biology to immune suppression and HCC outcome [13]. These studies suggest that the next chapter of awareness must include RNA biology. For hereditary cancer syndromes, in which germline lesions may affect DNA repair, chromatin control or signaling, non-coding RNA and post-transcriptional regulation may help explain variable penetrance, tumor heterogeneity and treatment response.

Multi-omics also moves hereditary cancer awareness from static risk to dynamic disease biology. A study of Cullin-family genes in colorectal cancer identified CUL5 and CUL7 as prognostic markers and contributors to progression, with evidence of epigenetic activation [14]. In esophageal squamous cell carcinoma, a succinylation-related prognostic model integrated transcriptomic, immune, miRNA-mRNA, protein-interaction and single-cell analyses [15]. In gastric cancer, histamine-associated prognostic genes were used to construct a risk model connected to metabolism and immune infiltration [16]. In NSCLC, miR-4652-3p was linked to inflammatory microenvironment-driven glutamine metabolism through the MYC/SLC1A5 pathway [17]. Such work reflects an important practical point: risk interpretation increasingly depends on models that integrate multiple biological dimensions. For hereditary cancer clinics, this could improve how we assess genetic modifiers, classify variants of uncertain significance, and use tumor profiling to guide germline counselling.

The tumor microenvironment is another essential layer. Hereditary cancer awareness often begins in the germline, but prevention and treatment unfold in tissues. Recent *Hereditas* studies on cancer-associated fibroblasts and immune escape in colorectal cancer, TLE1-associated osimertinib resistance and immune context in lung adenocarcinoma, and 3D cervical cancer models of therapy resistance each emphasize that cancer is an ecosystem [18–20]. Cells experiencing inherited or acquired vulnerabilities do not progress in isolation. They interact with fibroblasts, immune cells, extracellular matrix, oxygen gradients, nutrients and therapy-induced stress. For families carrying inherited risk, awareness must therefore include the concept that prevention is not only early detection of a lesion, but also early understanding of the environments that allow a lesion to persist, adapt and resist.

This is particularly important as hereditary cancer care increasingly intersects with precision oncology. A germline finding can influence therapy selection, surgical decisions and surveillance, but tumor evolution can still create resistance. *Hereditas* has recently published studies that connect computational discovery with experimental validation, including Pevonedistat targeting the CRL4-DTL-p21/p53 axis in nasopharyngeal carcinoma and ginkgetin-associated modulation of PI3K/AKT/mTOR signaling in bladder cancer [21, 22]. A review of 14–3–3ζ further emphasized the importance of signaling proteins in recurrence and chemoresistance [23]. These examples show that awareness should not be limited to genetic testing campaigns. It should also explain how genetic and multi-omic information can guide rational therapy, reveal resistance mechanisms and support survivorship.

The same principle applies to intervention at the level of gene regulation. Enhancer-based gene therapy, reviewed recently in *Hereditas*, illustrates how cell-type-specific regulatory elements, synthetic biology, delivery systems and genome editing may eventually support more precise modulation of disease pathways [24]. For hereditary cancers, this remains an emerging frontier rather than a routine clinical reality. Yet it changes the imagination of prevention. The future may not only involve finding people at risk earlier but also modifying risk-associated pathways with increasing specificity. Awareness must prepare society for this future while remaining honest about uncertainty, safety, access and ethics.

A multi-omics view also sharpens the ethical responsibilities of hereditary cancer awareness. More data do not automatically produce better care. Integrated genomics can widen disparities if testing, counselling, sequencing, computational expertise and follow-up are available only to selected groups. Multi-omics can also complicate communication: families already struggle with pathogenic variants, variants of uncertain significance, incidental findings and privacy concerns. Adding expression, methylation, immune and metabolic data will require better language, not more jargon. Awareness should therefore include data governance, equitable access, family communication, culturally sensitive counselling and realistic explanation of what multi-omics can and cannot currently do.

**Fig. 1:**
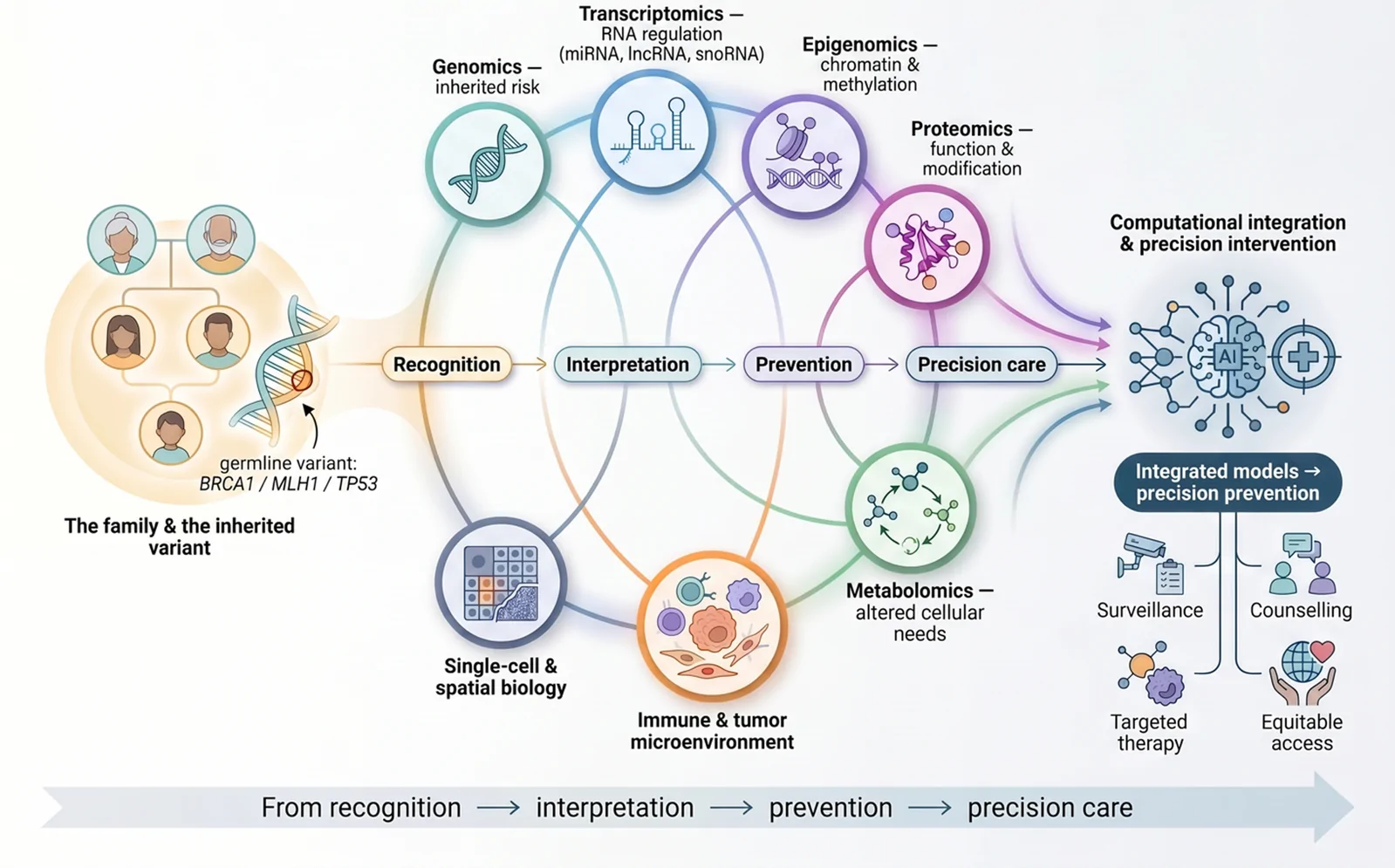
Hereditary cancer awareness is expanding from variant detection to multi-omics interpretation

*Hereditas* is well positioned to help define this standard. The journal’s recent cancer publications show breadth across miRNAs, lncRNAs, snoRNAs, epigenetic regulation, metabolism, immune biology, single-cell analysis, computational modelling, therapy resistance and precision intervention (Fig. 1). The task now is to connect this breadth explicitly to hereditary cancer awareness and to make the connection visible to authors, reviewers, clinicians, patients and advocacy communities. We should encourage studies that integrate germline, tumour and functional data; validate computational findings experimentally; report clinical relevance transparently; and consider how discoveries may support prevention, counselling and equitable implementation. In the multi-omics era, a journal does more than publish results; it curates the conceptual bridge between discovery and action.

Beyond the variant, therefore, is not simply a title. It is a call to update the public and professional meaning of hereditary cancer awareness. The variant remains the entry point: it identifies risk, guides relatives, and creates opportunities for prevention. But the future lies in understanding how those variants act within a living system. When awareness embraces multi-omics, it can move from recognition to interpretation, from interpretation to prevention, and from prevention to precision care. This is the next chapter for hereditary cancer awareness, and it is a chapter *Hereditas* can help write.
